# Pheochromocytoma/paraganglioma crisis: case series from a tertiary referral center for pheochromocytomas and paragangliomas

**DOI:** 10.1007/s42000-021-00274-6

**Published:** 2021-02-11

**Authors:** Anouk C Meijs, Marieke Snel, Eleonora P M Corssmit

**Affiliations:** 1 Department of Medicine, Division of Endocrinology, Leiden University Medical Center, Albinusdreef 2, 2333 ZA Leiden, The Netherlands; 2 Center for Endocrine Tumors Leiden (CETL), Leiden University Medical Center, Albinusdreef 2, 2333 ZA Leiden, The Netherlands

**Keywords:** Pheochromocytoma, Paraganglioma, Catecholamines, Metanephrines, Critical care medicine

## Abstract

Pheochromocytoma/paraganglioma (PPGL)-induced catecholamine crisis is a rare endocrine emergency leading to life-threatening hemodynamic instability causing end-organ damage or dysfunction. As it is associated with a significant mortality rate of approximately 15%, recognizing the signs and symptoms and making the appropriate diagnosis are critical. For this purpose, we report the clinical course of the crisis in four out of a total of six patients with a PPGL crisis from a cohort of 199 PPGL patients of a single tertiary referral center for PPGL patients in the Netherlands diagnosed between 2002 and 2020. Successful treatment of a PPGL crisis demands prompt diagnosis, vigorous pharmacological therapy, and emergency tumor removal if the patient continues to deteriorate.

## Introduction

Pheochromocytomas and paragangliomas (PPGLs) are rare neuroendocrine tumors arising from chromaffin cells of the adrenal medulla and the extra-adrenal neural crest progenitors, respectively, both of which may secrete catecholamines [1]. The estimated annual incidence of PPGL is 0.8 per 100,000 person-years [2], although PPGLs are more prevalent among patients with hypertension (0.1%) [3]. As clinical presentation is widely variable and nonspecific, diagnosing PPGL can be extremely challenging. Some patients present with the classic triad, which consists of episodic headache, diaphoresis, and tachycardia [4, 5], while others present with sustained or paroxysmal hypertension or with local tumor symptoms such as abdominal pain, or are asymptomatic [1]. Nevertheless, on rare occasions, patients present with a PPGL crisis, which has been defined as an acute severe presentation of catecholamine-induced hemodynamic instability causing end-organ damage or dysfunction [6]. The constellation of symptoms of a PPGL crisis can resemble other life-threatening conditions and can potentially be misdiagnosed as, e.g., septic shock, heart failure, thyroid storm, and malignant hyperthermia [1, 7]. As it concerns an endocrine emergency with a significant mortality rate of approximately 15%, recognizing the signs and symptoms of PPGL and making the appropriate diagnosis are critical [1, 8]. In this case series, four patients with a PPGL crisis are described with the aim of evaluating possible eliciting factors and the clinical course of the crisis.

## Patients and methods

In this retrospective single-center case series, medical records of patients operated on PPGL between 2002 and 2020 in the Leiden University Medical Center (LUMC), a tertiary referral center for PPGL in the Netherlands, were assessed for PPGL crisis. PPGL crisis was defined as acute and severe presentation of catecholamine-induced hemodynamic instability causing end-organ damage or dysfunction, associated with hypertensive crisis (systolic blood pressure >220 mmHg and/or diastolic blood pressure >120 mmHg) and/or hypotension (systolic blood pressure <90 mmHg and/or diastolic blood pressure <60 mmHg). Clinical characteristics including sex, age, pre-existing PPGL symptoms, mutation status, family history, crisis-precipitating factors, laboratory findings, radiologic imaging, clinical course, and treatment of the PPGL crisis were collected. Written informed consent was obtained from each participant after they were provided with a full explanation of the content of the study. The study was found to be exempt from consideration by the Medical Ethical Committee of Leiden University Medical Center.

## Results

Within a cohort of 199 patients who underwent surgery for a PPGL, six patients (3.02%) with PPGL crisis were identified from patient records. Four patients, of whom two have also been reported in earlier case studies [9, 10], gave written informed consent and were included. Two patients were not included as no informed consent was obtained due to the fact that one patient was lost to follow-up and one patient was deceased. The death caused by PPGL crisis of one out of six patients indicates a mortality rate of 16.7%.

Median age at presentation of the crisis was 43 years (range 17 to 50 years). Two patients were male. In three patients, the diagnosis of PPGL was established and subsequent alpha blockade using doxazosin was initiated before the crisis. Three patients had PPGL-related symptoms, including paroxysmal shortness of breath, palpitations, diaphoresis, pallor, nausea, chest pain, and orthostatic hypotension before the onset of the crisis. Three patients had sporadic pheochromocytoma and one patient a SDHB mutation with bone metastases of extra-adrenal abdominal paraganglioma. Tumor size varied between 6.5 and 13.4 centimeters. In one patient, the eliciting factor was a pathological fracture of large femoral bone metastasis, in another patient, it was a glucagon test, and in two patients, the eliciting factor remained unknown.

24-h urinary normetanephrine and metanephrine excess ranged from 3 to 35 times the upper limit of normal, plasma norepinephrine, and epinephrine excess reached as much as >100.000 nmol/L (Table 1). Other hormonal blood tests, including renin and aldosterone, did not show abnormalities of clinical significance.

**Table 1:** Clinical characteristics of patients with PPGL crisis

	Patient 1	Patient 2	Patient 3 [9]	Patient 4 [10]
Age at PPGL crisis	17	50	42	43
Gender	Male	Female	Female	Male
Genetic testing	SDHB mutation	Negative	Negative	Negative
Family history of PPGL	Negative	Negative	Negative	Negative
PPGL known before crisis	Yes	Yes	Yes	No
PPGL-related symptoms before crisis	No	Yes, paroxysmal shortness of breath, palpitations, diaphoresis, pallor, and nausea	Yes, shortness of breath, palpitations, diaphoresis, nausea, and chest pain	Yes, paroxysmal palpitations, chest pain, and orthostatic hypotension
Antihypertensive medication before crisis	Yes, doxazosin	Yes, doxazosin	Yes, doxazosin	No
Eliciting factor PPGL crisis	Pathological fracture large femoral bone metastasis	Unclear	Unclear	Glucagon test
Number of PPGL	1	1	1	1
Location of PPGL	Para-aortic caudal of the left kidney	Left adrenal medulla	Left adrenal medulla	Right adrenal medulla
Size of PPGL (cm)	6.9 × 6.5 × 4.9	9.8 × 10.3 × 13.4	6.5 × 5.0 × 6.0	9.4×7.9×8.3
Metastases	Yes, in bone	No	No	No
24-h urinary metanephrines				
Normetanephrine, μmol/mol creatinine (reference range)	2278 (25–280)	6.6 (0.6–2.0)	57.6 (0.0–3.3)	N.a.^a^
Metanephrine, μmol/mol creatinine (reference range)	114 (20–110)	54.6 (0.4–1.5)	0.9 (0.0–1.8)	N.a.
3-M-Tyramine, μmol/mol creatinine (reference range)	118 (20–200)	N.a.	3.0 (0.0–3.0)	N.a.
Plasma catecholamines				
Norepinephrine, nmol/L (reference range)	N.a.	N.a.	N.a.	>100.000 (0.95–3.11)
Epinephrine, nmol/L (reference range)	N.a.	N.a.	N.a.	>100.000 (0.17–0.61)

^a^*N.a.* not available

Blood pressure during crisis ranged from 88/63 (lowest) to 250/140 mmHg (highest). All four patients were admitted to the intensive care unit (ICU) and received respiratory support. Two patients were intubated and ventilated. Three patients had lactic acidosis and additional hyperglycemia. Cardiac crisis occurred in three out of four patients, including inverted Takotsubo and classic Takotsubo with and without hypertrophic obstructive cardiomyopathy (HOCM) with systolic anterior motion (SAM). No patient required extracorporeal membrane oxygenation (ECMO) or intra-aortic balloon pump (IABP). In two patients, continuous veno-venous hemofiltration (CVVH) was initiated in all patients pharmacological treatment consisted of alpha blockade either with phentolamine or with doxazosin, the latter in one hypotensive patient after initial hemodynamic stabilization with milrinone. Metyrosine was administered in two patients. In two patients, additional beta blockade was given using labetalol or metoprolol. Open surgery was performed in all patients, within a range of 18 to 32 days after the start of the crisis (Table 2).

**Table 2:** Clinical course and treatment PPGL crisis

	Patient 1	Patient 2	Patient 3 [9]	Patient 4 [10]
Hypertension, (highest blood pressure, mmHg)	No	Yes, 170/128	Yes, 225/140	Yes, 250/140
Hypotension, (lowest blood pressure, mmHg)	Yes, 88/63	No	No	Yes, exact value not known
Pulse rate, bpm	154	140	134	160
Temperature, °C	37.6	37.6	35.6	35.4
Crisis manifestations	Respiratory failure and hemodynamic instability due to inverted Takotsubo cardiomyopathy	Respiratory failure, renal failure, and hemodynamic instability with Takotsubo cardiomyopathy	HOCM^a^, SAM^b^, and Takotsubo cardiomyopathy	Respiratory failure, renal failure, and microangiopathic hemolytic anemia
Lactic acidosis	No	Yes	Yes	Yes
Hyperglycemia	No	Yes	Yes	Yes
Thrombosis	No	Possible clots in femoral vein due to puncture CVVH^c^	No	No
ICU admission	Yes	Yes	Yes	Yes
Respiratory support	Yes	Yes	Yes	Yes
Mechanic ventilation	No	Yes, in prone position	No	Yes, in prone position
ECMO^d^	No	No	No	No
IABP^e^	No	No	No	No
CVVH^c^	No	Yes	No	Yes
Pharmacological treatment	Milrinone, metyrosine, later switched to doxazosin	Milrinone, nitroglycerine, after diagnosis metyrosine and phentolamine, later switched to doxazosin	Nitroglycerine, metoprolol, after diagnosis switched to doxazosin and verapamil	Labetalol, phentolamine, and doxazosin
Surgery (incl. time between onset of the crisis and surgery)	Yes, 32 days	Yes, 18 days	Yes, ± 1 month	Yes, 31 days
Open or laparoscopic surgery	Open	Open	Open	Open
Complications during surgery	No	No	Yes, hypertensive crisis due to inadequate preoperative alpha blockade	No
Long-term complications	2017: Fracture femoral bone prosthesis for which reoperation 2019: Chronic infection prosthesis	No	Renal vein lesion during surgery resulting in atrophy left kidney	Chronic kidney insufficiency grade II

^a^*HOCM* hypertrophic obstructive cardiomyopathy

^b^*SAM* systolic anterior motion

^c^*CVVH* continuous veno-venous hemofiltration

^d^*ECMO* extracorporeal membrane oxygenation

^e^*IABP* intra-aortic balloon pump

Two illustrative cases, which have not previously been described in the literature, are described below.

### Patient 1

**Fig. 1:**
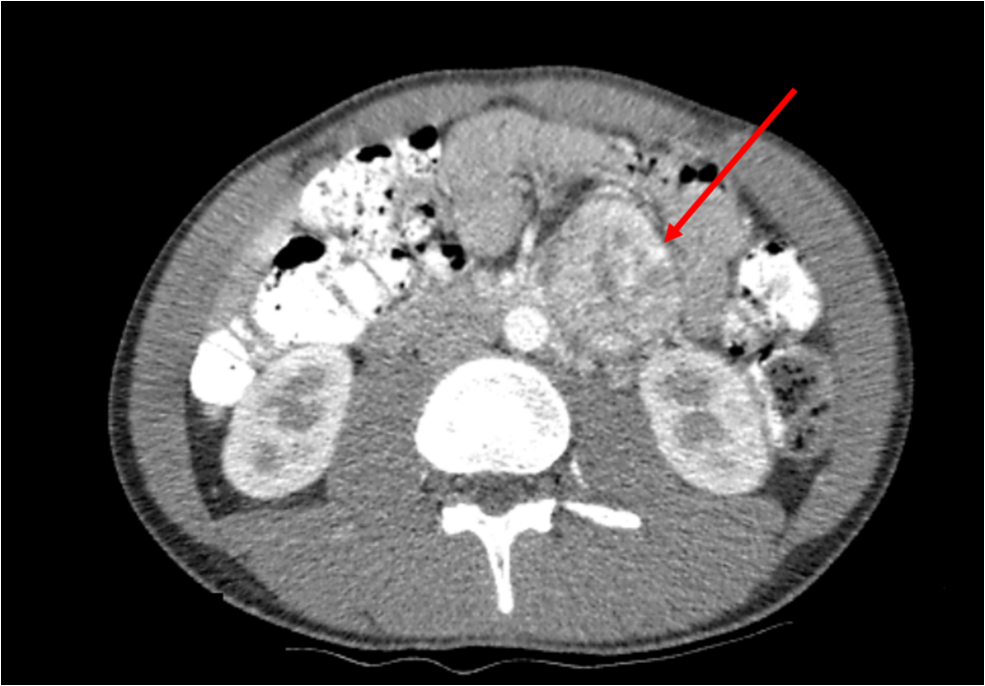
Abdominal CT scan demonstrating a hypervascular para-aortic mass of 7 cm with central necrosis, located caudal to the left kidney

In 2014, a 17-year-old man with unremarkable medical history was analyzed in the department of pediatric nephrology for hypertension (188/88 mmHg) combined with exercise-induced paroxysmal headaches. Unexpectedly, abdominal ultrasound revealed an inhomogeneous mass with a diameter of 6 cm. Abdominal MRI and CT scan confirmed a para-aortic mass of 6.9 × 6.5 × 4.9 cm caudal to the left kidney (Fig. 1) and disclosed multiple lytic bone lesions, suspicious for bone metastases. Additional ^123^I-metaiodobenzylguanidine (MIBG) scan showed that both abdominal mass and bone lesions were ^123^I-MIBG positive. Plasma normetanephrine concentration was highly elevated (21.35 nmol/L, reference range <1.07 nmol/L), plasma 3-M-tyramine slightly increased (0.27 nmol/L, reference range <0.17 nmol/L), and plasma metanephrine concentration in the normal range. Similar results were obtained from 24-h urine collections. A combination of clinical, laboratory, and imaging findings confirmed the diagnosis of paraganglioma with bone metastases.

**Fig. 2:**
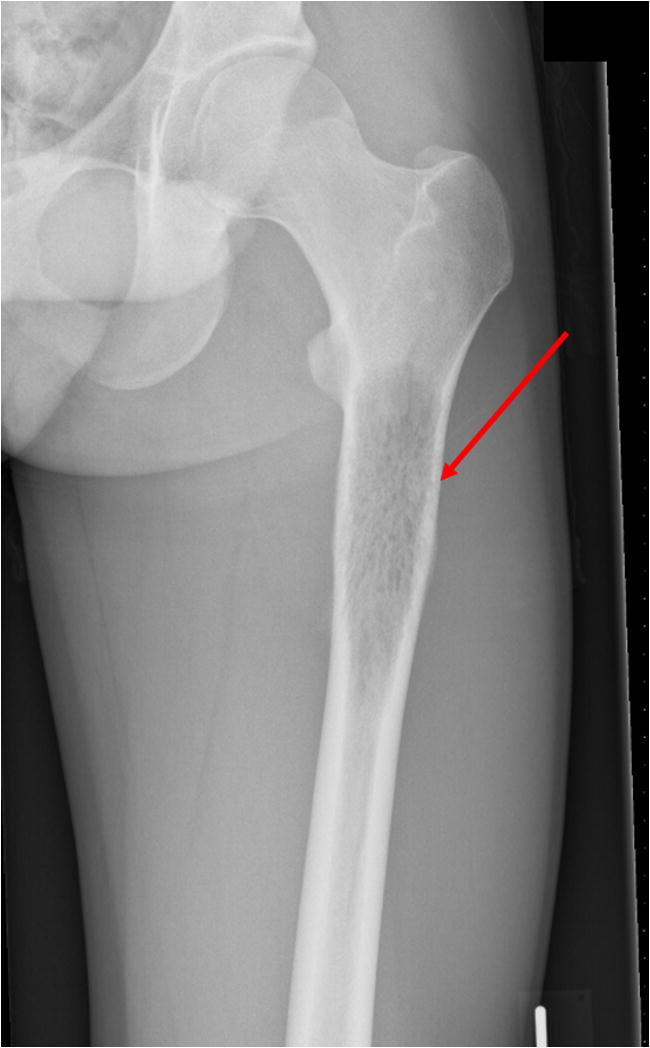
X-ray of the left femur demonstrating destruction of left femoral cortex due to bone metastasis

After preoperative alpha blockade, resection of the paraganglioma was performed. The pre- and postoperative course was uncomplicated. A few months later, radiologic imaging showed a residue of 1.0 × 0.7 cm in the location of the resected tumor, which was interpreted as a resorbing hematoma, and slow growth of the 2.6 × 2.6 × 11.1-cm lytic bone metastasis in the left femur, with cortex destruction, causing increased fracture risk (Fig. 2). Therefore, orthopedic surgery to stabilize the femur was scheduled.

However, a few weeks before the scheduled surgery, the patient fractured his left femur at the site of the known lytic lesion and soon afterwards developed hypoxia (oxygen saturation 80% at room air), tachycardia (154 beats per min (bpm)), and hypotension (88/63 mmHg). Further vital parameters included body temperature of 37.6 °C and respiratory rate of 20 breaths per min. Arterial blood gas analysis revealed metabolic acidosis combined with respiratory acidosis; lactate levels were normal. Additional abnormal laboratory results included an increased troponin level (3.020 μg/L, reference range <0.050 μg/L). Chest radiography suggested a consolidation in the right lower lobe, which was interpreted as a possible fat embolism due to the pathologic fracture and lack of infection parameters. Electrocardiogram (ECG) showed sinus tachycardia of 150 bpm with normal conduction and repolarization. Emergency surgery was contraindicated as the patient was hypotensive, and alpha blockade could not be initiated. Therefore, the femur was immobilized, after which the patient was admitted to the ICU for respiratory support using optiflow and hemodynamic support. Echocardiography showed akinetic basal and mid-ventricular segments of the left ventricle, interpreted as inverted Takotsubo cardiomyopathy. Therapy with milrinone, a phosphodiesterase inhibitor, was started, after which hemodynamic stability was reached. In addition to milrinone, metyrosine was initiated to reduce catecholamine synthesis, resulting in further improvement of his clinical condition and recovery of systolic function on echocardiography. Milrinone was stopped and doxazosin started in anticipation of orthopedic surgery of his femoral bone, which was performed 32 days after the onset of the crisis.

Although his family history was negative, genetic testing for known germline mutations was positive for SDHB mutation, confirming the diagnosis of hereditary paraganglioma/pheochromocytoma syndrome type 4 (PGL4).

### Patient 2

In 2017, a 50-year-old female patient, with a medical history including multiple sclerosis and NSTEMI, was admitted to the hospital with acute shortness of breath. The patient had been referred to the Endocrinology Outpatient Clinic approximately 2 months earlier for evaluation of an accidentally discovered 9.8 × 10.3 × 13.4-cm left-sided adrenal lesion on CT. In retrospect, this mass had also been detectable on MRI performed 11 years earlier, at that time being 4.7 cm in diameter and interpreted as a cyst. Retrospectively, the patient had longstanding symptoms of paroxysmal shortness of breath, palpitations, diaphoresis, pallor, and nausea, previously attributed to hyperventilation. Blood pressure was normal (120/88 mmHg). 24-h urinary excretion of metanephrine and normetanephrine was markedly increased (54.6 μmol/mol creatinine, reference range 0.4–1.5; and 6.6 μmol/mol creatinine, reference range 0.6–2.0, respectively).

Additional imaging showed positive MIBG-uptake in the left adrenal mass but no metastases. The combined clinical, laboratory, and imaging findings were consistent with the diagnosis of a functional pheochromocytoma of the left adrenal gland, after which alpha blockade with doxazosin was initiated.

Unexpectedly, before elective adrenalectomy was scheduled, the patient developed acute nausea and shortness of breath in the presence of hypertension (blood pressure 170/128 mmHg), tachycardia (pulse rate 140 bpm), and hypoxia (oxygen saturation 80% at room air). Laboratory examination demonstrated lactic acidosis, compromised liver and renal function, and slightly elevated troponin, while chest X-ray showed pulmonary edema in both lower lobes. Echocardiography revealed decreased left ventricle function (LVEF 29%) and left ventricular wall motion abnormalities, which had significantly deteriorated compared to the findings of the echocardiography performed 3 weeks earlier. No significant coronary stenosis was objectivated and ECG remained unchanged.

The patient was transferred to the ICU for closer monitoring. The differential diagnosis consisted of acute cardiac forward and backward failure due to sepsis or catecholamine crisis. The patient was intubated and ventilated in the prone position. In addition, CVVH was initiated as she developed severe metabolic acidosis due to acute kidney failure. Pharmacological treatment consisted of furosemide, nitroglycerine, (nor)adrenalin, milrinone, and terlipressin. In addition, cefuroxime and gentamicin were administered to cover for a possible infection. As acute coronary syndrome (ACS) could not be not excluded, acetylsalicylic acid, clopidogrel, and enoxaparin were also given.

After a few days, bacterial cultures remained sterile and it was concluded that the signs and symptoms were caused by a pheochromocytoma multisystem crisis with secondary Takotsubo cardiomyopathy. Consequently, (nor)adrenalin and cefuroxime were discontinued. The patient was started on metyrosine combined with phentolamine. When oral intake was possible, phentolamine was switched to doxazosin.

As the patients’ clinical status improved after a few more days, including increase of LVEF to 53%, surgery was delayed until she was completely stable and total alpha blockade was reached. Eighteen days after the onset of the crisis, open adrenalectomy was performed. The pre- and postoperative course was uncomplicated. To date, the patient has not relapsed. Family history and genetic testing for known germline mutations were negative.

## Discussion

In this case series, four patients with a PPGL crisis are described. We reveal an incidence of PPGL crisis of 3.02% among PPGL patients in a single tertiary referral center for PPGL patients in the Netherlands. The incidence we report is lower than the incidence of 7–18% reported by other studies [11–13]; a recent multicenter retrospective review by Riester et al. reported an incidence of catecholamine crisis of 11% in PPGL patients [11], while two other retrospective studies in patients who underwent surgery for PPGL found an incidence of 18 and 7% [12, 13]. The difference between our and other studies might be related to the high prevalence of germline mutations, especially in succinate dehydrogenase (SDH) genes, in the Netherlands. It has been reported that in 1045 DNA samples of Dutch PPGL patients, 690 were mutation-positive (66%) [14], suggesting a higher prevalence of syndromic PPGL compared to those noted in other studies, which reported a prevalence of syndromic PPGL in their cohorts ranging from 14 to 21% [11–13]. Syndromic PPGL patients are offered annual clinical evaluation and regular biochemical screening for metanephrine excess as well as radiological imaging for PPGL, which might result in a relatively high detection rate of asymptomatic and early-stage PPGL. It has been reported that patients screened for PPGL because of hereditary predisposition had lower urinary excretion rates of catecholamines and smaller tumors than patients presenting with symptomatic PPGL [15], possibly resulting in better accessible surgical resection of the smaller PPGLs and less perioperative complications, such as crises.

The mortality rate revealed in our case series was 16.7%, which is comparable to the mortality rate of 15% reported by Whitelaw et al. [1].

PPGL crisis is thought to be related to a sudden increase in catecholamine release. However, the underlying pathophysiological mechanism leading to this surge in catecholamine release is not as yet fully understood. Nevertheless, a wide variation of precipitating factors has been postulated, including physical stimulus to the tumor [1], as occurred in patient 1 of our case series who developed a PPGL crisis after fracturing his left femur at the site of a large bone metastasis. Administration of glucagon which stimulates secretion of catecholamines by the pheochromocytoma, as happened in patient 4, has also previously been described as an eliciting factor for PPGL crisis [16]. The most prevalent precipitating factor is surgical resection of the PPGL. Other well-established precipitating factors include insufficient tumor blood supply by tumor hemorrhage or infarction, general anesthesia, drugs (including dopamine antagonists, beta-blockers without prior alpha blockade, steroids, and antidepressants), and pregnancy [1, 17].

Not only are precipitating factors variable, but also presentation of PPGL crisis differs markedly, as the various catecholamines and the relative proportions of the type of catecholamine excreted cause different effects [18]. Nevertheless, the type of catecholamine excess does not seem to predict the clinical course of the crisis [1]. The usually abrupt but nonspecific symptoms of PPGL crisis often raise diagnostic challenges. The variation in the clinical picture regularly results in suspicion of another diagnosis, most frequently sepsis [6]. In turn, the diagnostic delay causes postponement of adequate treatment, resulting in worse outcome. Therefore, in any patient presenting with unexplained shock or left ventricular failure, multiple organ failure, hypertensive crisis, or unexplained lactic acidosis, diagnosis of PPGL crisis should be considered (Table 3) [1]. Initial biochemical testing for PPGLs should include measurements of plasma free metanephrines or urinary fractionated metanephrines. Once there is clear biochemical evidence of a PPGL, imaging studies should be performed to locate the PPGL [17].

**Table 3:** Signs and symptoms suspicious for PPGL crisis

Diagnosis of PPGL (before crisis)	
PPGL-related symptoms before crisis (e.g., paroxysmal headache, diaphoresis, and palpitations)	
Unexplained shock • Bacterial cultures remaining sterile in patients suspicious for septic shock (multiple organ failure, fever) • No coronary stenosis in patients with cardiogenic shock suspicious for myocardial infarction (raised troponin, acute left ventricular failure, low cardiac index)	
Multiple organ failure: cardiac, respiratory, renal, hepatic, neurological, gastrointestinal, metabolic, vascular, and/or musculoskeletal manifestations	
Hypertensive crisis	
Labile blood pressure: alternating hyper- and hypotension	
Unexplained lactic acidosis	

A diagnostic challenge is particularly illustrated in patient 2, who had paroxysmal symptoms including palpitations, diaphoresis, and pallor for 14 years and an adrenal mass of 4.7 cm detected by MRI and interpreted as a cyst 11 years before the correct diagnosis was made. Despite the fact that three patients had been diagnosed with a PPGL and surgery had been scheduled, crisis could not be prevented.

In three out of four patients, PPGL crisis included cardiac involvement, which is one of the most commonly reported complications of a PPGL crisis [19]. Cardiac crisis is caused by catecholamine excess, which induces artery vasoconstriction and vasospasm. This may result in myocardial ischemia and, potentially, infarction in the absence of clinically significant atherosclerotic coronary disease. Additionally, catecholamines have a direct toxic effect on myocytes, which causes catecholamine cardiomyopathy [1]. The classic presentation is Takotsubo cardiomyopathy, characterized by the rapid development of severe, left ventricular dysfunction involving the mid-ventricular and apical segments, in the absence of obstructive coronary artery disease [1, 20]. Patient 1 developed a variant of this syndrome called inverted Takotsubo, which can be distinguished from classic Takotsubo by dysfunction of the basal and mid-ventricular segments with preservation of apical segments [20]. Takotsubo cardiomyopathy typically presents with signs and symptoms consistent with acute myocardial infarction, including chest pain and shortness of breath, ECG changes of ST-segment elevation or depression later evolving to diffuse T-wave inversions, and elevated cardiac enzymes. The development of left ventricular dysfunction can cause acute complications, including dysrhythmias, pulmonary edema, and cardiogenic shock. Fortunately, ventricular function is almost universally reversible [20], which was also observed in our cases. Nevertheless, prognosis depends on the duration of catecholamine exposure and the extent of myocardial damage [21]. A recent review of 59 acute cardiomyopathy cases related to PPGL revealed that these patients most frequently had mixed epinephrine and norepinephrine excess [22]. In our case series, one of three patients with cardiac involvement had mixed epinephrine and norepinephrine excess and the other two patients had exclusively normetanephrine excess.

Besides cardiac involvement, excessive secretion of catecholamines may affect numerous other organ systems. Catecholamines predominantly act on alpha-adrenergic receptors, causing arterial vasoconstriction resulting in hypertension and relatively reduced intravascular volume. Consequently, reduced end-organ perfusion and tissue ischemia may develop, which can result in respiratory, renal, hepatic, neurological, gastrointestinal, metabolic, vascular, or musculoskeletal manifestations [1]. In addition, uncontrolled hypertension has been shown to cause endothelial injury and destruction of red blood cells, resulting in microangiopathic hemolytic anemia [23], as occurred in patient 4.

Evidence for treatment of PPGL crisis is based on expert opinion, case reports, and retrospective series. Although some general concepts are necessary, treatment should be tailored to each patient based on the type of organ system involved and the extent of the crisis. Whitelaw et al. [1] proposed a sub-classification of PPGL crisis distinguishing limited type A from extensive type B crisis. They defined type A as hemodynamic instability and end-organ damage in one or more organs, which could potentially progress to type B, i.e., an extensive crisis with sustained hypotension, shock, and multiple organ dysfunction (involvement of two or more organ systems). Minimally invasive monitoring of cardiac index and oxygen levels and treatment with alpha blockade may be appropriate in type A crisis. However, in type B crisis, more extensive monitoring, including pulmonary artery catheter combined with frequent or continuous transesophageal cardiac ultrasound, might be indicated. This permits real-time assessment of left ventricular function, including filling status, left ventricular outflow tract dimensions, and regional wall abnormalities [1]. Additionally, management of type B crisis might be challenging since alpha blockade, the standard therapy in PPGL crisis, cannot be initiated as it aggravates hypotension. Besides volume resuscitation as conventional supportive treatment, mechanical support, including IABP and ECMO, have successfully been used in these patients. This mechanical support might allow for alpha blockade sooner and, thereby, result in better outcomes [24]. A recent systematic review including 62 patients supported with extracorporeal life support because of intractable pheochromocytoma crisis showed that in-hospital survival was 87%, indicating a lower mortality rate than the previously reported mortality rate of 20% in extensive type B crisis [1, 25].

As in PPGL crisis patients already have high catecholamine levels, vasoactive amines should be avoided. Besides, these patients might be relatively insensitive to the action of vasoactive amines due to alpha and beta receptor downregulation after long-term exposure to high levels of catecholamines [24]. It has been suggested that inodilators, such as milrinone and levosimendan, which act through non-adrenergic pathways, could be good alternatives to provide inotropic support and relieve afterload. Although the European Society of Cardiology guidelines do not recommend milrinone in the treatment of Takotsubo cardiomyopathy, there are multiple PPGL cases described in the literature in which it has been successfully used, including one of our hypotensive patients [26–30].

Intensive intravenous fluid resuscitation is needed in both type A and type B crisis. Crystalloid intravenous fluids should be given prior to or concurrently with alpha blockade, as patients have relative intravascular hypovolemia which may not be clinically apparent until alpha blockade is administered. Hemodynamic monitoring and monitoring of oxygenation gives direction to the speed and adequacy of fluid resuscitation [1].

After stabilization, alpha blockade is the standard of care. Phenoxybenzamine is most commonly used [31]. However, doxazosin, which can only be administered orally, is becoming more popular due to the selective blockade of the alpha-1 receptors, resulting in fewer side effects. Moreover, it has been demonstrated that doxazosin has similar efficacy to that of phenoxybenzamine [17, 32]. Other pharmacological treatment options include calcium channel blockers and magnesium sulfate, which acts as a functional calcium antagonist and reduces the secretion of catecholamines [1, 33]. Beta-blockers can be used after adequate alpha blockade to control reflex tachycardia or tachydysrhythmia [1]. Furthermore, metyrosine can be given in addition to alpha blockade to reduce catecholamine synthesis by inhibiting tyrosine hydroxylase, which catalyzes tyrosine to dihydroxyphenylalanine (DOPA), the first and the rate-limiting step of the catecholamine synthesis pathway [34]. It has been suggested that the addition of metyrosine results in better blood pressure management than alpha blockade alone [35, 36].

**Fig. 3:**
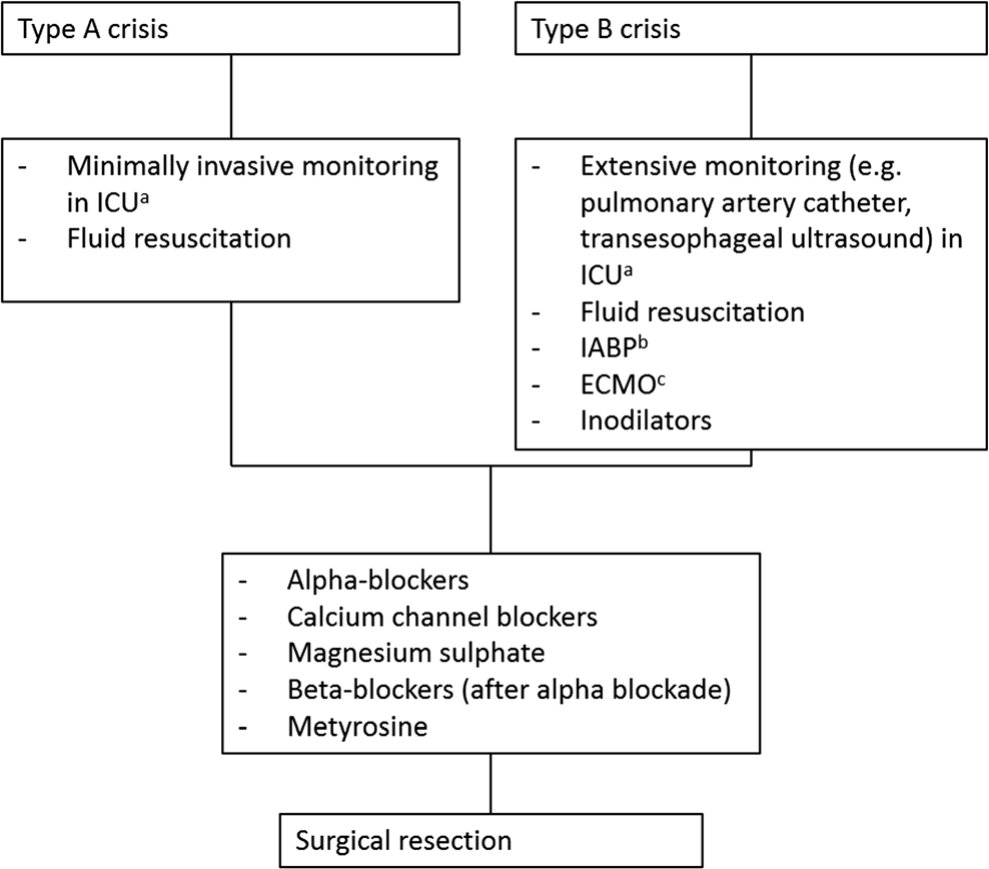
Treatment in patients with PPGL crisis. Type A crisis: hemodynamic instability and end-organ damage in one or more organs. Type B crisis: sustained hypotension, shock, and multiple organ dysfunction (two or more organ systems). Abbreviations: ^a^ICU intensive care unit, ^b^IABP intra-aortic balloon pump, ^c^ECMO extracorporeal membrane oxygenation

Surgery provides the only curative treatment for PPGL [3]. As illustrated in patient 3, who developed intraoperative hypertensive crisis due to initiation of surgery before adequate alpha blockade was accomplished, optimal timing of surgery and preoperative treatment are crucial. It has been proposed that emergency surgery is indicated in patients who fail to reach medical stabilization, as removing the source of catecholamine excess might enable rapid recovery. In addition, deferring surgery puts the patient at risk for developing recurrent crisis, with further organ dysfunction [1]. Whitelaw et al. recommended that medical stabilization should be attempted prior to surgery, unless factors such as tumor rupture and hemorrhage occur. However, the safety of deferring surgery in deteriorating patients remains to be elucidated [1] (Fig. 3)*.*

In conclusion, we report four patients with PPGL crisis presenting with divergent clinical phenotypes with the aim of increasing awareness of the diagnosis among clinicians and of evaluating the clinical course and precipitating factors. As PPGLs frequently mimic other conditions, it is important to consider PPGL crisis in any patient with unexplained shock or left ventricular failure, multiple organ failure, hypertensive crisis, or unexplained lactic acidosis especially if the patient is also febrile. Timely recognition and confirmation of the diagnosis followed by optimal management are essential to improve survival. Although some common approaches to management are necessary, specific treatment decisions need to be tailored to each patient based on the extent of the crisis and which organ systems are involved [1]. The present description of the management of our patients may provide direction to clinicians concerning treatment options for patients with this rare presentation of PPGL.
